# Cryptococcal pathogenic mechanisms: a dangerous trip from the environment to the brain

**DOI:** 10.1590/0074-02760180057

**Published:** 2018-04-12

**Authors:** Shannon K Esher, Oscar Zaragoza, James Andrew Alspaugh

**Affiliations:** 1 Duke University School of Medicine, Department of Molecular Genetics and Microbiology, Department of Medicine, Durham, USA; 2 Instituto de Salud Carlos III, National Centre for Microbiology, Mycology Reference Laboratory, Madrid, Spain

**Keywords:** Cryptococcus neoformans, capsule, melanin, intracellular pathogen, metabolic adaptation, dissemination

## Abstract

*Cryptococcus neoformans* is an opportunistic pathogenic yeast that causes serious infections, most commonly of the central nervous system (CNS). *C. neoformans* is mainly found in the environment and acquired by inhalation. It could be metaphorically imagined that cryptococcal disease is a “journey” for the microorganism that starts in the environment, where this yeast loads its suitcase with virulence traits. *C. neoformans* first encounters the infected mammalian host in the lungs, a site in which it must choose the right elements from its “virulence suitcase” to survive the pulmonary immune response. However, the lung is often only the first stop in this journey, and in some individuals the fungal trip continues to the brain. To enter the brain, *C. neoformans* must “open” the main barrier that protects this organ, the blood brain barrier (BBB). Once in the brain, *C. neoformans* expresses a distinct set of protective attributes that confers a strong neurotropism and the ability to cause brain colonisation. In summary, *C. neoformans* is a unique fungal pathogen as shown in its ability to survive in the face of multiple stress factors and to express virulence factors that contribute to the development of disease.

Invasive fungal infections are estimated to cause over 1.5 million deaths per year worldwide, with the vast majority of these infections occurring in immunocompromised patients (Brown et al. 2012). Over the last few decades, the emergence of HIV infection in particular has led to the rise in cases of cryptococcal meningoencephalitis. Caused by the basidiomycete yeast *Cryptococcus neoformans,* cryptococcosis globally results in approximately 215,000 infections per year, leading to 180,000 deaths patients (Rajasingham et al. 2017). *C. neoformans* can be isolated from the environment in many regions of the world, resulting in nearly universal exposure to this fungus among human populations. However, symptomatic disease after exposure is relatively rare. Defects in cell-mediated immunity, especially as directed by CD4+ lymphocytes, are the most common risk factors for developing invasive cryptococcal disease. Additional predisposing factors include solid organ or bone marrow transplantation-associated immunosuppression, treatment with corticosteroids, treatment with tumor necrosis factor-a inhibitors, various malignancies, sarcoidosis, chronic liver disease, and renal failure (Casadevall and Perfect 1998, Baddley et al. 2008, Maziarz and Perfect 2016). Cryptococcosis is a common AIDS-defining illness and a leading cause of mortality among adults with HIV in Sub-Saharan Africa (Rajasingham et al. 2017). Despite the advent of antiretroviral therapy, which drastically reduced the number of HIV cases in the developed world, *C. neoformans* remains a major problem in resource-limited regions. Furthermore, while the number of AIDS-associated cases of cryptococcal disease has decreased overall, the incidence of disease in solid organ transplant patients and other non-AIDS-associated cases has increased (Bratton et al. 2012).

Although this fungus is found primarily in the environment, it possesses features that allow survival and proliferation within a human host. Moreover, *C. neoformans* must be able to move from the lungs, the most common initial site of infection, to the central nervous system (CNS), the most common site of symptomatic disease. To accomplish this journey, *C. neoformans* has developed inducible and highly regulated cellular processes that favor fungal survival despite formidable host defenses.

## CRYPTOCOCCUS IN THE ENVIRONMENT AND ACQUISITION OF INFECTION: GETTING THE SUITCASE READY AND FULL OF VIRULENCE ATTRIBUTES

*C. neoformans* is frequently found in the environment in association with pigeon guano, as well as in association with a variety of trees and soils (Emmons 1955, Litvintseva et al. 2011, Chowdhary et al. 2012). While *C. neoformans* does not generally cause symptomatic disease in pigeons due to their high body temperature, these birds are thought to be a reservoir contributing to the global dispersion of this pathogen (Littman and Borok 1968, Litvintseva et al. 2011). *C. neoformans* is found throughout the world and can infect a wide variety of hosts, including cats, dogs, koalas, dolphins, and even plants (Lester et al. 2004, McGill et al. 2009, Kido et al. 2012, Venn-Watson et al. 2012, Pennisi et al. 2013, Warpeha et al. 2013). Studies estimate that 50% of urban children have been exposed to *Cryptococcus* by age 2 (Goldman et al. 2001). A related *Cryptococcus* species, *Cryptococcus gattii* is found in more restricted geographic regions. Unlike *C. neoformans, C. gattii* is found almost exclusively in association with certain plant species, including eucalyptus trees (Ellis and Pfeiffer 1990). Infections with *C. gattii* have some overlapping features with those due to *C. neoformans*; however, *C. gattii* tends to cause disease more often in people without clear immunodeficiencies. Additionally, *C. gattii* infections often present with focal brain abscesses, instead of more generalised CNS infections (Mitchell et al. 1995, Speed and Dunt 1995, Byrnes et al. 2011). Interestingly, murine inhalation models of *C. gattii* infections demonstrate that this *Cryptococcus* species tends to more frequently cause focal pulmonary disease rather than dissemination to the CNS. These clinical and experimental data suggest that *C. gattii* may possess microbial features that favor localised and tissue-specific infections rather than widespread systemic dissemination (Ngamskulrungroj et al. 2012).

*C. neoformans* has several well characterised virulence attributes, including the ability to grow at mammalian body temperature and the production of polysaccharide capsule, melanin, urease, and phospholipases. *C. neoformans* has also developed several strategies to survive and replicate within phagocytic cells. However, despite these successful virulence strategies, *C. neoformans* does not require the mammalian host to complete its lifecycle, leading to its designation as an “accidental pathogen” (Casadevall and Pirofski 2007). Instead, it has been hypothesised that these virulence traits were acquired for survival in the environment, and then re-purposed in the setting of mammalian infection. For example, the antiphagocytic capsule has been hypothesised to protect cells from environmental desiccation. Similarly, the antioxidant melanin pigment, which is required for survival *in vivo,* is thought to shield against UV radiation-induced cellular damage (Aksenov et al. 1973, Nosanchuk and Casadevall 2006).

It has also been suggested that *C. neoformans* may have evolved to survive encounters with free-living soil microbes, such as amoebae and nematodes (Steenbergen and Casadevall 2003, Casadevall and Pirofski 2007). Amoebae including *Acanthamoeba castellanii* and *Dictyostelium discoideum* can interact and ingest *C. neoformans* in a manner similar to mammalian macrophages (Steenbergen et al. 2001). *C. neoformans* is able to kill these organisms, and many of the virulence attributes described above were also shown to be important for survival within amoebae (Steenbergen et al. 2001). Similar protozoa, as well as bacteria and insects, have been isolated from pigeon guano and shown to influence *C. neoformans* growth (Ruiz et al. 1982). Interestingly *C. neoformans,* but not related non-pathogenic cryptococcal species, can kill the nematode *Caenorhabditis elegans* (Mylonakis et al. 2002). Interaction with *A. castellanii*, as well as the wax moth *Galleria mellonella*, induced *C. neoformans* capsule and the formation of giant/titan cells, similar to what has been observed in mammalian models of infection (Chrisman et al. 2011, García-Rodas et al. 2011).

*Role of spores* - *C. neoformans* isolated from the environment grows almost exclusively as a haploid budding yeast. This asexual form replicates by mitosis. *C. neoformans* also has a defined sexual cycle in which it grows in a filamentous form. Mating occurs when partners of opposite mating types *(MATa* and *MATa)* fuse and form filaments with distinct nuclei and specialised clamp cells. The dikaryotic filaments eventually produce a basidium, a terminally differentiated structure at the end of a growing hyphae, in which nuclear fusion and meiosis occur to produce chains of haploid a and a basidiospores at the basidial head (Kwon-Chung 1976). Strains of the a mating type can also undergo monokaryotic haploid fruiting or same sex mating (Wickes et al. 1996, Lin et al. 2005). Interestingly, most clinical and environmental isolates are exclusively a mating type, leading to questions about the frequency of sexual reproduction in nature (Kwon-Chung and Bennett 1978). However, Litvintseva et al. (2003) identified fungal populations in Botswana in which the proportion of MATa and MATa isolates is relatively even. Further analysis revealed evidence of clonal expansions and recombination among this population (Litvintseva et al. 2003, Chen et al. 2015).

It has long been debated whether desiccated yeasts or spores are the predominant infectious particles of *C. neoformans*. Despite a lack of evidence for frequent sexual reproduction as a mechanism to generate infectious basidiospores, monokaryotic haploid fruiting can potentially result in the production of abundant spores in the absence of a mating partner. While a liquid suspension of fungal cells is generally used in murine models of cryptococcal infection, several studies have demonstrated that spores are capable of producing infection (Sukroongreung et al. 1998, Giles et al. 2009, Velagapudi et al. 2009, Springer et al. 2013). Detailed spore analyses have been delayed due to difficulties in purifying large numbers of spores to homogeneity. However, recent advances in spore isolation have led to the characterisation of spore morphology, stress tolerance, and surface/coat composition (Botts et al. 2009). These studies revealed that the spore surface is composed of specialised polysaccharides, which are thought to aid in persistence in the environment (Botts et al. 2009). Recent comparative proteomic analyses highlighted proteins involved in spore composition as well as proteins important for spore germination and initiation of vegetative growth (Huang et al. 2015).

The recognition of spores compared to yeast-like cells by the immune system has also been investigated, revealing important differences in how these two morphological states are sensed (Giles et al. 2009, Walsh et al. 2017). Unlike yeast cells, spores are readily phagocytosed by macrophages, inside which they can germinate and replicate. One caveat, however, is that activated macrophages can rapidly kill ungerminated spores, which are highly susceptible to ROS (Giles et al. 2009). Therefore, the ability for spores to produce an active infection is dependent on their ability to germinate prior to macrophage activation and killing. Once germinated, the budding yeast cells can grow both intracellularly, and extracellularly in the host, prompting investigators to refer to this microbe as a facultative intracellular pathogen.

## FIRST STOP OF THE TRIP: ADAPTATION TO THE LUNG ENVIRONMENT (CHOOSING THE RIGHT TRAIT FROM THE VIRULENCE SUITCASE)

A key step in the trip of the cryptococcal disease is its arrival at the lungs. The immune response of this organ is complex and specialised because it is frequently exposed to a large number of exogenous particles suspended in the air, like dust and microorganisms. For this reason, this organ has complex and specialised immune responses to control the continuous challenge from external threats. One of the main mechanisms of defense in the lung depends on tissue-resident macrophages present in the alveoli, which phagocytose and remove exogenous particles and microorganisms. In addition, the lung contains the surfactant system, which is a mixture of phospholipids and glycoproteins whose main function is to maintain the superficial tension during respiration. Some of these surfactant proteins also have antimicrobial properties, as they can bind to microbes and induce phagocytosis.

After inhalation, the infectious particles of *C. neoformans* have to evade this complex immune response and replicate. Survival in this environment is not an intrinsic property of most microbes. In fact, in animal models, most fungi cannot cause lung infection. For example, the immune response of the lung typically results in complete clearance of most *Candida* species. Even in the case of *Aspergillus fumigatus,* a filamentous fungus that can cause pulmonary infection in immunosuppressed individuals, colonisation of the mouse lungs only occurs when the animals are immunosuppressed. For this reason, *C. neoformans* is a remarkable fungal pathogen due to its effective evasion of the lung immune system, and in fact, it behaves like other primary fungal pathogens, such as *Histoplasma* or *Paracoccidioides* species. In the next sections, we will briefly describe the virulence factors and adaptation mechanisms elicited by *C. neoformans* that produce its adaptation to the lung.

*Metabolic adaptation to temperature, nutrients and metals* - While there are over 1.5 million fungi, only a handful of these are capable of growing at elevated temperatures, including human body temperature (37°C). Within the basidiomycetes, pathogenic *Cryptococcus* species are the only organisms known to grow well at high temperatures (Perfect 2006). It has been demonstrated that growth at 37°C can protect against the accumulation of deleterious mutations, suggesting a role for this trait in genomic stability beyond contributing to pathogenesis in a mammalian host (Xu 2004). Considered one of the main virulence attributes of this organism, many investigators have worked to characterise the proteins important for tolerance to high temperature. Components of the mitochondrial antioxidant response, including manganese superoxide dismutase, have been shown to be important for high temperature growth, as well as virulence (Giles et al. 2005). Trehalose, a sugar made by fungi and not by mammals, protects *C. neoformans* from internal and external stresses, including high temperature. Components of the trehalose biosynthesis pathway are required for high temperature growth as well as virulence in a variety of infection models (Petzold et al. 2006). A number of *C. neoformans* signal transduction pathways also have major roles in sensing and responding to high temperature stress. These include the calcium/calmodulin/calcineurin pathway (Odom et al. 1997, Kraus and Heitman 2003), MAP kinase pathways including the PKC/cell wall integrity pathway (Kraus et al. 2003, Gerik et al. 2005, Gerik et al. 2008) and the high osmolarity glycerol (HOG) response pathway (Bahn and Kojima 2005, Bahn et al. 2007), and the Ras signaling pathway (Alspaugh et al. 2000). Aside from their roles in thermotolerance, these pathways contribute to the fungal response to other stress responses, and each plays a central role in virulence.

In addition to adapting to the high temperature of the mammalian host, *C. neoformans* must also adapt to limitations and/or influxes of essential nutrients and metals. Analysis of the *C. neoformans* genes expressed in the context of murine infected lungs showed the up-regulation of many genes involved in carbon metabolism (Hu et al. 2008). Hu and colleagues also found that various transporters, including those for monosaccharides, acetate, iron, and copper, were all induced in the murine lung (Hu et al. 2008). Similarly, a study of the transcriptomes of two clinical isolates from human CSF demonstrated that upregulated genes were enriched for GO terms associated with cellular metabolism in these *in vivo* clinical samples compared to *ex vivo* incubated samples (Chen et al. 2014).

Iron availability is an important aspect of cryptococcal pathogenesis, and detailed studies have explored the role of this metal in various aspects of its physiology. *C. neoformans* and other microbes compete with the host for iron, and iron sequestration is a basic component of host “nutritional immunity”. This metal is required for both capsule and melanin synthesis, and excess iron can contribute to exacerbated meningoencephalitis in mouse models of infection (Barluzzi et al. 2002). *C. neoformans* has several enzymes and transporters that aid in the acquisition of iron from the host (reviewed in Jung and Kronstad 2008). Under the transcriptional control of the central iron regulator, Cirl, *C. neoformans* possesses many cell surface proteins that facilitate iron uptake into the fungal cell. These surface proteins include iron reductases that reduce extracellular iron to allow transport into the cell, iron permeases such as Cft1, and plasma membrane ferroxidases such as Cfo1 to convert iron atoms to biologically optimised oxidation states.

*C. neoformans* must also be able to sense and respond to the essential metal copper. Copper is both simultaneously required and detrimental for *C. neoformans* growth *in vivo*. Copper is an important cofactor for a number of enzymatic reactions, in addition to being required for the enzymatic activity involved in melanin formation. However, there is increasing evidence that it is used by the host as a microbicide; innate immune cells upregulate copper importers to accumulate copper in the phagosome (White et al. 2009). Furthermore, alveolar cells isolated from mice challenged with *C. neoformans* were shown to have increased expression of copper importers and higher levels of intracellular copper (Ding et al. 2013). As with iron, much work has gone into defining the proteins and enzymes required for the response to and regulation of copper uptake and overload. The transcription factor Cuf1 has been shown to regulate the response to both high and low copper conditions (Waterman et al. 2007, Ding et al. 2011). In the lungs and the phagosomes of innate immune cells, *C. neoformans* experiences high copper conditions, in which Cuf1 directs the upregulation of the copper-detoxifying metallothioneins, *CMT1* and *CMT2,* and downregulates the expression of copper importers (Ding et al. 2013, Sun et al. 2014). In contrast to its mutational state while in the lung, *C. neoformans* experiences low copper conditions during brain infection during which Cuf1 directs the transcriptional induction of the *CTR1* and *CTR4* copper importers, among other proteins to control copper homeostasis (Ding et al. 2013, Sun et al. 2014).

Upon transitioning to the host environment, *C. neoformans* must also adapt to the relatively alkaline pH of the mammalian lung. Changes in ambient pH can induce stress on many important cellular processes, including nutrient uptake, protein stability and function, and membrane and cell wall stability and maintenance. The Rim alkaline response pathway is the main signaling pathway responsible for sensing and responding to changes in external pH (reviewed in Selvig and Alspaugh 2011). Activation of the pathway occurs when alkaline pH is sensed at the cell surface by the membrane sensing complex composed of the Rra1 membrane protein and members of the ESCRT machinery (Hu et al. 2013, Hu et al. 2015, Ost et al. 2015). The assembled ESCRT complexes serve as a scaffold for the proteolysis complex composed of Rim20, Rim23, and the Rim13 protease, which cleaves the Rim101 transcription factor so that it can transit to the nucleus to regulate gene expression (O’Meara et al. 2010, O’Meara et al. 2014, Ost et al. 2015). Rim101 directly regulates genes required for various stress responses including low iron and elevated salt concentrations (O’Meara et al. 2014). It is also required for proper formation of the polysaccharide capsule and proper cell wall maintenance in response to host conditions (O’Meara et al. 2010, O’Meara et al. 2013, O’Meara et al. 2014, Ost et al. 2017).

*Adaptation to free radicals: melanin and antioxidant mechanisms* - Once inside the phagosome, *C. neoformans* must also adapt to reactive oxygen species (ROS) in order to survive within this environment. Melanin is perhaps the most well-known factor involved in ROS tolerance. In *C. neoformans,* melanin synthesis depends on laccase enzymes, which uses dopaminergic precursors, mainly L-DOPA to produce the pigment. In fact, both laccase activity and the accumulation of melanin pigments are required for pathogenesis (Kwon-Chung and Rhodes 1986, Williamson 1994). Melanin in *C. neoformans* accumulates at the cell wall and confers resistance to many different types of stresses (Nosanchuk and Casadevall 2003). Melanised *C. neoformans* strains were less susceptible than melanin deficient strains to nitrosative and oxidative stresses (Wang and Casadevall 1994). As a free radical scavenger, melanin is capable of neutralising ROS (Jacobson and Hong 1997). Additionally, laccases, the enzymes responsible for making melanin, interfere with the oxidative burst of phagocytes in part by sequestering and oxidising iron during infection (Jacobson and Hong 1997, Liu et al. 1999). Melanin and the Lac1 laccase enzyme have also been demonstrated to facilitate dissemination of *C. neoformans* from the lung to the CNS (Noverr et al. 2004).

In addition to melanin, combined proteomic and genetic analyses have identified several other cellular processes involved in the nitrosative stress response, from canonical cellular stress response pathways to cell wall maintenance, signal transduction, intracellular transport, transcriptional control, respiration, and metabolism (Missall et al. 2006). Other classical enzymes, including copper- and zinc-containing superoxide dismutase and components of the thioredoxin and glutathione antioxidant systems, have been highlighted in the response to oxidative and nitrosative stresses, as well as in promoting fungal virulence (Cox et al. 2003, Missall and Lodge 2005a, Missall and Lodge 2005b, Missall et al. 2005). Surprisingly, catalases, enzymes that detoxify hydrogen peroxide, were shown not to play a major role in ROS stress tolerance in *C. neoformans,* perhaps due to functional redundancy with other elements of ROS defense (Giles et al. 2006).

*The polysaccharide capsule* - The most characteristic feature of *C. neoformans* is its capsule, a complex network of polysaccharides present around the cell wall. This structure has been extensively studied for decades, but there are still key aspects about its biology that remain unknown. The capsule is not required for the replication of the yeast in regular laboratory conditions, as acapsular mutants can divide as well as wild type strains. However, the capsule is very important for virulence (Fromtling et al. 1982, Chang and Kwon-Chung 1994). Prior studies have demonstrated that the polysaccharide capsule contributes to disease in two complementary ways. First, it confers a protective shield to the yeast against the multiple challenges produced by the immune system. Additionally, its components exert a large number of deleterious effects on the host (reviewed in Vecchiarelli 2000, Zaragoza et al. 2009, O’Meara and Alspaugh 2012, Vecchiarelli and Monari 2012). For this reason, the capsule is considered the main virulence factor of this yeast.

*Capsular composition and capsule organisation* - The capsule is mainly composed of two complex polysaccharides: glucuronoxylomannan (GXM) and glucuronoxylomannogalactan (GXMGal) (Bose et al. 2003, Janbon 2004, Heiss et al. 2009). In turn, GXM is composed of a chain of mannose residues with substitutions of xylose and glucuronic acid. In the case of GXMGal, the main component is a chain of galactose molecules with substitutions of mannose, xylose and glucuronic acid. Many of the proteins and enzymes involved in the synthesis of the capsule have been defined (reviewed in Doering 2009), but there are still important aspects that remain uncharacterised. Although it is known that the polysaccharide capsule is organised as interwoven fibres (Pierini and Doering 2001, McFadden et al. 2007, Frases et al. 2009), the mechanisms by which these fibres are assembled remain to be elucidated. Interestingly, there is strong evidence to indicate that the capsule polymers are branched, forming micro-gel like structures (Cordero et al. 2011, Araújo et al. 2016). In addition to being present on the cell surface, capsular polysaccharides can also be found in extracellular vesicles (EVs) (Rodrigues et al. 2007). These structures have therefore been proposed as a mechanism for the extracellular export of capsule components, both for targeting to the cell surface and for release into the surrounding environment. It is still unknown how EVs are formed and trafficked to the outer surface of the cell, allowing release and attachment of capsule components. There are many genes required for capsule polysaccharide synthesis (reviewed in Doering 2000, Bose et al. 2003, O’Meara and Alspaugh 2012), however a large proportion of these genes still have uncharacterised functions.

*The capsule as a protective structure* - Before and during the interaction with the host, the presence of a capsule confers resistance to multiple types of stress. For example, it protects the fungal cell against environmental challenges such as dehydration (Aksenov et al. 1973). Furthermore, some of its roles are required during an actual infection. During infection the capsule contributes to evasion of phagocytosis-mediated killing by alveolar macrophages through several mechanisms. First, it impairs the recognition of cell wall epitopes by macrophage receptors, contributing to phagocyte avoidance (Kozel and Gotschlich 1982). In addition, the capsular polysaccharides have antioxidant properties, protecting the fungal cell from the toxic effects of reactive oxygen species produced in the phagolysosome (Zaragoza et al. 2008).

*Changes in capsular size and structure as mechanisms of immune evasion* - The capsule is a dynamic structure that changes its composition, structure, and size depending on the environmental conditions. Among these phenomena, one of the best studied is the change in size. The capsule diameter is normally small during growth in rich media, however there is a significant increase in its size after interaction with the host (Feldmesser et al. 2001). This enlargement has been described during infection in animal models and phagocytic cells (Chrisman et al. 2011), the non-vertebrate host *G. mellonella* (García-Rodas et al. 2011), and even environmental predators such as free-living amoebas (Chrisman et al. 2011). Furthermore, there are several factors that induce this transition *in vitro,* such as CO_2_ (Granger et al. 1985), iron limitation (Vartivarian et al. 1993), mammalian serum (Zaragoza et al. 2003a) and nutrient limitation (Zaragoza and Casadevall 2004). This process seems to be important from a clinical point of view, since there is a correlation between *ex vivo* capsule size and the intracranial pressure of patients affected by cryptococcal meningoencephalitis (Robertson et al. 2014). Capsule enlargement poses a significant change for the cells and it is believed that it is an energy-costly process that requires protein synthesis and the correct functioning of mitochondria (Trevijano-Contador et al. 2017). During infection, capsule enlargement confers resistance to complement-mediated phagocytosis (Zaragoza et al. 2003b) and contributes to killing-avoidance in macrophages (Zaragoza et al. 2008). Cells with larger capsules are also more resistant to oxidative stress, antimicrobial peptides and antifungal compounds (i.e., amphotericin B).

The capsule also can undergo other rearrangements that have profound consequences for pathogenesis and immune evasion. For example, the structure and organisation of the polysaccharide fibres can substantially change in the host. There are several monoclonal antibodies (mAbs) to the capsule available, and the binding properties of these mAbs to *C. neoformans* cells obtained from *in vivo* samples is variable, even changing during the course of infection (Garcia-Hermoso et al. 2004). These dynamic capsular changes result in a very heterogeneous population of cryptococcal cells that differ in their epitope composition, which impairs the effectiveness of a proper immune response. Furthermore, changes in capsule structure have been also related dissemination efficiency and to brain invasion (Garcia-Hermoso et al. 2004). In addition, the structure of the capsule can undergo microevolution *in vitro*, making the microbial population phenotypically and antigenically variable in laboratory cultures depending on the growth conditions (McFadden et al. 2006).

Finally, the density of the polysaccharide fibers also increases *in vitro* (Maxson et al. 2007) and during infection (Gates et al. 2004). Although the consequences of this increase in density are not fully known, it produces a capsular structure that is less permeable to elements of the immune response, such as antibodies, complement or antimicrobial peptides.

*Exopolysaccharides as virulence factors* - The polysaccharides of the capsule are not only attached to the cell, but they are also released into the medium (exopolysaccharides). During infection, extracellular capsular polysaccharides can be found in tissues, CSF, and blood. Recent work has demonstrated that the release of exopolysaccharides is a regulated process in *C. neoformans* that depends on environmental cues and distinct genes (Denham et al. 2017). These polysaccharides seem to contribute to the development of disease through multiple mechanisms. Among them, both GXM and GXMGal can cause apoptosis of several types of immune cells through activation of FasL/Fas (Chiapello et al. 2003, Monari et al. 2005b, Monari et al. 2006, Monari et al. 2008, Villena et al. 2008). Secreted polysaccharides can also impair Ab production, induce complement depletion (Macher et al. 1978), inhibit leukocyte migration (Dong and Murphy 1995, Dong et al. 1999, Ellerbroek et al. 2002), reduce immune cell infiltration to the brain (Denham et al. 2017), and stimulate the production of cytokines and chemokines (Monari et al. 2005a, Vecchiarelli et al. 2011). Furthermore, these polysaccharides are recognised by several types of immune receptors, such as CD18, CD14 and toll-like receptors (TLRs) (Shoham et al. 2001, Taborda and Casadevall 2002, Yauch et al. 2004).

*Intracellular survival inside macrophages/recognition by macrophages -* Upon entering the lung, one of the first cell types that *C. neoformans* engages are innate immune phagocytes, in particular alveolar macrophages. *C. neoformans* has a dynamic relationship with macrophages, and there is data to support their role in both clearance and persistence of this fungus. For example, depletion of macrophages reduces survival in murine models of infection (Monga 1981). On the other hand, while classical virulence attributes such as capsule and melanin assist the fungus to minimise phagocytosis and killing by macrophages, *C. neoformans* requires macrophages for efficient dissemination to the CNS (Charlier et al. 2009).

*Recognition* - The interaction between fungi and host begins when fungal factors are recognised by innate immune cell surface receptors, triggering immune cell activation and inducing phagocytosis of the fungus. A number of pattern recognition receptors (PRRs) recognise *C. neoformans*, including receptors in the Toll-like (TLR), C-type lectin (CLR), and NOD like families (NLR), as well as scavenger receptors. Acapsular strains are readily ingested by phagocytosis through interactions with the mannose receptor (MR) and Dectin-1 (Cross and Bancroft 1995, Casadevall and Perfect 1998, Heitman et al. 2010). Capsule components can also be recognised by several receptors, including TLR2, TLR4, and the co-receptor CD14 (Shoham et al. 2001, Yauch et al. 2004, Yauch et al. 2005). While there is opposing evidence as to the importance of TLR2 in the immune response to *C. neoformans*, it is clear that TLR4 is not required for protection against *C. neoformans* in mouse models of infection (Yauch et al. 2004, Biondo et al. 2005, Nakamura et al. 2006). A major role for MyD88 (the adaptor protein that directs downstream immune signalling from many of the TLRs) has been demonstrated by multiple groups; mice that are deficient in MyD88 succumb to *C. neoformans* infection at rates significantly faster than WT mice (Yauch et al. 2004, Biondo et al. 2005).

The CLR Dectin-2, which recognises mannan in the fungal cell wall, is associated with higher levels of non-protective Th2 cytokines during *C. neoformans* infection (Nakamura et al. 2015). While Dectin-1 can bind to b-glucan on *C. neoformans* spores (Giles et al. 2009), its role in phagocytosis of spores as well as overall protection against *C. neoformans* infection appears to be minimal (Nakamura et al. 2007, Walsh et al. 2017). Dectin-3 deficiency was also shown not to be a major factor in immunity towards *C. neoformans* (Campuzano et al. 2017). However, mice deficient in the CLR adaptor protein Card9, were highly susceptible to *C. neoformans* infection due to decreased influx of INF-g producing cells, suggesting a role for CLR-mediated signalling pathways in protection from cryptococcal infection (Yamamoto et al. 2014). Finally, both the mannose receptor (MR) and DC-SIGN recognise mannosylated proteins on the *C. neoformans* cell surface (Mansour et al. 2006), and MR-deficient mice are highly susceptible to infection with *C. neoformans* (Dan et al. 2008). Together these data suggest that a combination of immune receptors might act in hetero-complexes to recognise the dynamic surface of *C. neoformans*, leading to complex downstream immune signalling, similar to what has been described for recognition of other fungal species (reviewed in Inoue and Shinohara 2014).

While capsule and cell wall components can be recognised by several PRRs, encapsulated strains require opsonisation with antibodies or complement for efficient phagocytosis. Anti-capsular antibodies can be recognised by CD19 and Fcg receptors (Netski and Kozel 2002). The localisation of the antibody binding, as well as antibody isotype, impact the efficiency of phagocytosis (Nussbaum et al. 1997, Cleare and Casadevall 1998). The cryptococcal capsule is also capable of inducing complement activation through the alternative pathway. This activation results in the deposition of complement proteins within the capsule structure (Kozel 1996), which can be recognised by CD11b/CD18 and CD11c/CD18 (Taborda and Casadevall 2002). Similar to antibody-mediated phagocytosis, the efficiency of complement-mediated phagocytosis depends on capsule size and location of complement protein binding (Kozel 1996, Zaragoza et al. 2003b, Zaragoza et al. 2009). Importantly, complement-deficient animals were more susceptible to *C. neoformans* infection (Rhodes 1985).

*Phagocytosis* - As a facultative intracellular pathogen, *C. neoformans* has many strategies to regulate its phagocytosis by immune cells. Perhaps the best studied is the polysaccharide capsule, which itself inhibits phagocytosis by macrophages (Bolanos and Mitchell 1989, Levitz and DiBenedetto 1989). The specific capsule components can also influence its interaction with host cells through differential binding of opsonins as described above (Kozel et al. 1988, Zaragoza et al. 2003b).

In addition to capsule, Luberto and colleagues identified an antiphagocytic protein, App1, that has an important role in phagocytosis and virulence in *C. neoformans*. Importantly, this protein was identified in the serum of AIDS patients with disseminated *C. neoformans*, highlighting its physiological importance (Salgado et al. 1994, Luberto et al. 2003). *In vitro,* treatment of cells with App1 inhibited engulfment in a complement-dependent manner (Stano et al. 2009). Conversely, *applΔ* cells were more readily phagocytosed and displayed attenuated virulence in multiple mouse backgrounds (Luberto et al. 2003, Del Poeta 2004). Similarly, there is another regulator, Gat201, which mediates phagocytosis avoidance through a capsule-independent mechanism (Liu et al. 2008).

*Survival and proliferation inside macrophages* Despite actively avoiding phagocytosis, *C. neoformans* is quite capable of surviving and proliferating inside of phagocytic immune cells. In fact, *C. neoformans* is viable and replicates within the acidic environment of the phagolysosome (Levitz et al. 1999, Qin et al. 2011). Additionally, phagosomes containing *C. neoformans* experience lysosomal fusion and acquire phagosomal markers, indicating that phagosomal maturation is not inhibited by this pathogen (Coelho et al. 2014). More recent work has demonstrated that several of these early markers are prematurely removed and that *C. neoformans* can subtly alter the phagosome maturation process in order to create a more permissive environment for its survival (Smith et al. 2015). Through a screen to identify host factors that influence intracellular survival, it was shown that *C. neoformans* hijacks many aspects of macrophage biology, including cytoskeletal elements, cell surface signaling molecules, and vesicle mediated transport systems, to favor its own survival (Qin et al. 2011). This study also showed that autophagy proteins are recruited to pathogen-containing vacuoles, supporting *C. neoformans* infection (Qin et al. 2011). Once ingested, *C. neoformans* induces phagolysosomal damage (Feldmesser et al. 2000, Tucker and Casadevall 2002, Davis et al. 2015), perhaps as a result of the combination of increased cell/capsule growth and secreted *C. neoformans* proteins, such as phospholipase B (Feldmesser et al. 2000, Cox et al. 2001). The damaged phagolysosomes display increased membrane permeability which enhances *C. neoformans* growth by allowing nutrient influx, pH homeostasis, and eventually escape from the macrophage (Davis et al. 2015).

*Macrophage exit* - Once inside of a macrophage, *C. neoformans* has several potential fates. The first is being inhibited or killed by the phagocyte. Other options, all of which ultimately lead to fungal escape, include lysis of the macrophage, cell-to-cell transfer to a neighbouring macrophage, and non-lytic exocytosis or “vomocytosis” in which both fungal cell and macrophage survive the interaction (Johnston and May 2013, Coelho et al. 2014, De LeónRodríguez and Casadevall 2016). Lateral transfer of *C. neoformans* from one macrophage to another, while a rare event, allows for fungal cells to disseminate while avoiding immune detection. Alvarez and Casadevall (2007) demonstrated that this occurs in an actin-dependent manner, leaving lasting effects on the inhabited macrophage in the form of a large residual vacuole. This process can occur regardless of serotype or opsonisation type, and in multiple mammalian cell lines (Ma et al. 2007).

Non-lytic exocytosis, or vomocytosis, is similar to cell-to-cell spread in that both the fungal and macrophage cells are viable after fungal escape. This process has been shown to occur *in vivo* and is dependent on several host factors (Nicola et al. 2011). It appears to occur after phagosome maturation and is influenced by phagosomal pH. For example, when the pH of the phagosome was raised artificially with weak bases, rates of vomocytosis increased (Ma et al. 2006, Nicola et al. 2011). Concordantly, when acidification of the phagosome was blocked altogether using vacuolar ATPase inhibitors, the rate of vomocytosis decreased (Ma et al. 2006, Nicola et al. 2011). Phagosomal membrane permeabilisation occurs rapidly after uptake of *C. neoformans* cells and is thought to be another contributing factor to rates of non-lytic exocytosis (Tucker and Casadevall 2002, Coelho et al. 2014, Davis et al. 2015). Actin flashes around the phagosome occur soon after membrane permeabilisation and contribute to blocking non-lytic exocytosis (Johnston and May 2010). It has also been demonstrated that cytokine signalling has an impact on this process, with Th2-stimulated macrophages having lower rates of non-lytic exocytosis (Voelz et al. 2009). In addition to host factors, *C. neoformans* proteins are also required for non-lytic exocytosis, including phospholipase B1 (Plb1) and the Sec14 protein required for phospholipase secretion (Chayakulkeeree et al. 2011).

## Morphological changes in *C. neoformans and their* role during adaptation to the host

*Hyphal formation* - Many fungi undergo morphological changes during various stages of an infection, such as the transition among *Candida* species from a yeast-like form to hyphae and pseudohyphae. These filamentous structures are more adherent than blastoconidia, so they are involved in attachment, invasion and dissemination (reviewed in Trevijano-Contador et al. 2016). In the case of *C. neoformans,* this yeast can only form hyphae during sexual reproduction (Casadevall and Perfect 1998), and true hyphae are not believed to significantly contribute to the development of the disease. In contrast, there are other types of morphological changes that can occur in the host that are more relevant to our understanding of the pathogenesis of this microorganism. For example, *C. neoformans* can form pseudohyphae and they can be occasionally observed *in vivo* (Lee et al. 2012, Magditch et al. 2012), although their exact function in the adaptation of this yeast to the host remains unknown.

*Titan cells* - Although filamentous forms can be found in the host, the most well characterised mechanism developed by *C. neoformans* to adapt to the lung environment is its ability to increase its cells size. In fact, a significant feature of the cryptococcal population *in vivo* is its size heterogeneity, finding cells *in vivo* of very different diameters. Cellular enlargement can be achieved not only by capsule growth (which has been described above), but also by a significant increase in the size of the cell body, which leads to the appearance of rounded yeast cells of an abnormal size that can reach up to 100 microns (Okagaki et al. 2010, Zaragoza et al. 2010). These forms have been termed titan cells due to their huge size (Zaragoza and Nielsen 2013). The signals that induce the massive cellular enlargement are unknown. The main intracellular pathway involved in this process depends on cAMP and PKA signaling (Zaragoza et al. 2010), and several effectors upstream (such as pheromone receptors and Gpr5) and downstream (Rim101) are required for cell growth (Okagaki et al. 2011). As a consequence, there are alterations in cell cycle regulation that result in genome endoduplication and polyploidy (Okagaki et al. 2010, Zaragoza et al. 2010). In addition, the capsule of these cells is also very large and composed of a net of polysaccharide fibres that form a structure that is denser then that observed with cells of normal size (Zaragoza et al. 2010).

The role of titan cells in cryptococcal disease remains to be fully elucidated, however, their involvement in several processes that contribute to immune evasion and long-term persistence has been demonstrated. Titan cells cannot be phagocytosed presumably due to their size, as it was demonstrated that similarly sized synthetic particles could not be readily engulfed by lung phagocytes (Okagaki and Nielsen 2012). Interestingly, titan cells are also able to confer this phagocytosis resistance to neighbouring, smaller yeast cells (Okagaki and Nielsen 2012). The exact mechanism by which titan cells are able to provide collateral protection to neighbouring cells has not been precisely defined. However, it has been demonstrated that polyploid cells can produce a variety of haploid and aneuploid daughter cells, promoting rapid adaptations to stress conditions (Gerstein et al. 2015). Given the extensive surface capsule of titan cells, it is also plausible that secreted exopolysaccharide may influence the surrounding environment.

The signals that trigger titan cell production are unknown. It was first described that co-infection of mice with opposing mating type cells resulted in a significant increase in the proportion of titan cells (Okagaki et al. 2010), suggesting that the pheromone signalling pathway is required for this transition. Furthermore, titan cell production is strongly dependent on the host environment, and the percentage of these cells observed *in vivo* varies in different mouse strains. In particular, in mice that develop a Th1 type response (dependent on interferon-y and TNF-α), the proportion of titan cells is low (around 15%). In contrast, in mice that induce Th2 type responses, the proportion of titan cells is very high, even above 50% of the total population of cryptococcal cells (García-Barbazán et al. 2016). At the moment, the exact correlation between the host immune response and cryptococcal morphology is unknown, but it is hypothesised that Th2 type responses result in a less aggressive environment that facilitates cellular enlargement.

*Cell wall rearrangements during infection* - In addition to these well-characterised morphological changes, there is increasing evidence that *C. neoformans* cell wall maintenance plays an important role in its interaction with the host immune system. *C. neoformans* dramatically alters its cell wall, both in size and composition in response to the host environment (Feldmesser et al. 2001, O’Meara et al. 2013, O’Meara et al. 2014, Ost et al. 2017). Feldmesser et al (2001) demonstrated that the cell wall thickens over time in the setting of murine pulmonary infection. Additionally, both capsule and titan cell formation involve significant cell wall remodelling; polysaccharide capsule attaches to the cell surface through an interaction with a-(1,3)-glucan (Reese and Doering 2003), and titan cells have thicker, more chitin-rich cell walls (Wiesner et al. 2015). Studies investigating the Rim101 transcriptome during murine infection indicated that this pH responsive transcription factor directly regulates cell wall biosynthesis genes in this context (O’Meara et al. 2013, O’Meara et al. 2014). Coordinately, in the absence of Rim101, *C. neoformans* cells expose immunogenic epitopes that ultimately lead to detrimental immune responses (Feldmesser et al. 2001, O’Meara et al. 2013, O’Meara et al. 2014). These studies highlight how *C. neoformans* actively remodels its cell surface in response to the host environment in order to avoid immune detection.

## THE TRIP CONTINUES: DISSEMINATION THROUGH THE ORGANISM AND ARRIVAL TO THE BRAIN. DIVING OR SAILING?

Although cryptococcal cells are mainly acquired by inhalation, a key step in disease is the dissemination from the lung to the brain, where it causes the most characteristic clinical manifestation of cryptococcal disease, meningoencephalitis. For this reason, the mechanisms of migration of *C. neoformans* to the brain have been extensively studied (see seminal review in Griffiths et al. 2012). This dissemination occurs through the blood vessels, so cryptococcal cells must cross both epithelial and endothelial barriers to transit from the lung alveoli to the bloodstream, and ultimately the CNS.

The first barrier that *C. neoformans* faces during dissemination is composed of the epithelial cells from the lung, although this interaction has not been characterised in detail. It has been described that both encapsulated and acapsular cells can interact with human lung epithelial cells, with acapsular mutants being able to recognise and attach to this epithelial layer with greater affinity (Merkel and Scofield 1997). As a result, *C. neoformans* cells can be internalised by epithelial cells, leading to the death of the host cell. In the case of regular encapsulated cells, the capsular polysaccharide, GXM, plays a major role in the recognition by epithelial cells, and this binding seems to depend on the CD14 receptor. In addition, other cryptococcal proteins, such as the mannoprotein MP84 or phospholipase B, also seem to contribute to epithelial cell binding (Ganendren et al. 2006, Teixeira et al. 2014).

Of particular interest is the interaction of *C. neoformans* with endothelial cells, particularly those comprising the blood-brain barrier (BBB). Although the BBB selectively protects the brain from extracellular particles, *C. neoformans* has developed ways to cross this restrictive barrier, both as free-living fungal cells and intracellularly inside macrophages. Elegant real-time *in vivo* imaging experiments have demonstrated that isolated fungal cells can directly attach to the endothelial surface of the brain microvasculature as the initial step in breaching the BBB (Shi et al. 2010). Cryptococcal cells can subsequently be internalised by the endothelial cells at the apical side and then released at the basolateral side (Chen et al. 2003). In this process, it has been shown that hyaluronic acid (HA) present in *C. neoformans* can be recognised by the CD44 receptor from endothelial cells, suggesting a process of endothelial cell interaction that his conserved among several microbial neuropathogens (Jong et al. 2008). Interestingly, inositol produced by the host cells is recognised by *C. neoformans*, a process that results in an increased production of HA by the fungus (Liu et al. 2013). Other cryptococcal elements, such as urease (Olszewski et al. 2004), phospholipase B (Santangelo et al. 2004), and the extracellular protease Mpr1 (Na Pombejra et al. 2017) have been shown to be involved in the process of binding to endothelial cells. In this last case, the Mpr1 protease induces cytoskeleton rearrangements in the endothelial cells and promotes recognition of *C. neoformans* by Annexin A2. Internalisation of cryptococcal cells by the BBB is also associated with multiple changes in the endothelial cells including rearrangements of the cytoskeleton and changes in the morphology of nuclei, endoplasmic reticulum and mitochondria (Vu et al. 2013).

*C. neoformans* can also alter the structure of the tight junctions that attach the cells of the BBB (Olszewski et al. 2004, Charlier et al. 2005, Vu et al. 2013), so it has been suggested that *C. neoformans* can also transverse the BBB through a paracellular mechanism. In this sense, some addictive drugs (such as methamphetamine) that alter the structure of the BBB tight junctions increase the dissemination of cryptococcal cells to the brain (Eugenin et al. 2013).

There is also strong evidence that *C. neoformans* can cross the BBB inside phagocytic cells, through a process that is widely known as the “Trojan-horse” dissemination mechanism. This idea was first suggested when it was found that *C. neoformans* can survive inside phagocytic cells. In the last few years there has been increasing evidence that this dissemination mechanism occurs *in vivo.* Several elegant studies have demonstrated that macrophages have a paradoxical role during infection because their depletion has a protective role during cryptococcosis and results in reduced fungal burden in brain, lung and spleen (Kechichian et al. 2007, Charlier et al. 2009), suggesting that in fact these phagocytic cells offer a “safe” niche for this fungus and contribute to dissemination. In agreement, when mice are injected with bone marrow-derived monocytes infected with *C. neoformans,* the fungal burden in target organs is higher compared to infection with the equivalent dose of free living yeasts (Santangelo et al. 2004, Charlier et al. 2009). Further evidence has been provided *in vitro* using models of reconstituted BBB. In these experiments, *C. neoformans* can transmigrate across an *in vitro*-generated BBB via transcellular pores when transported inside macrophages (Sorrell et al. 2016, Santiago-Tirado et al. 2017). Santiago-Tirado et al. (2017) further demonstrated that, during this process, several outcomes of the interaction of *C. neoformans* and macrophages occur, such as fungal replication, non-lytic exocytosis and cell-to-cell transmission of fungal cells. In addition, these authors also observed direct transmission of cryptococcal cells from macrophages to endothelial cells, which suggests that the same fungal cell can transmigrate the BBB through several mechanisms (“Trojan horse” approach for dissemination through the blood stream, and paracellularly through endothelial cells as free cells).

In summary, there is strong evidence that *C. neoformans* can disseminate and colonise the brain through different mechanisms, although at the moment it is not known the relative contribution of each mechanism (as free yeasts or inside phagocytic cells). Due to the importance of this process for cryptococcal disease, further work is required to characterise this process and envision therapeutic strategies to control brain invasion by this fungus.

## FINAL STOP OF THE TRIP: SURVIVAL WITHIN THE CNS

Although survival in the lung and dissemination are key aspects to understand cryptococcal disease, the mechanisms that allow survival in the brain are also very important to define since the most common clinical manifestation of cryptococcal disease is brain infection (Colombo and Rodrigues 2015). Once *C. neoformans* has invaded the CNS, the clinical manifestations of the real tissue (meningitis) as well as from involvement of the brain tissue itself (encephalitis). Therefore, the symptoms of cryptococcal meningoencephalitis can range from a progressive headache to serious neurological symptoms, including coma and death. Moreover, the viscous capsular polysaccharide of this microorganism can trigger increased intracranial pressure, a major source of morbidity in this infection that must be treated aggressively.

Several investigators have explored how this organism is able to survive in the nutrient-poor environment of the cerebrospinal fluid, as well as in the specialised neural tissue. *C. neoformans* is able to grow *in vitro* on a very minimal medium composed primarily of cerebrospinal fluid (Chen et al. 2014). This fluid has a low carbohydrate and nitrogen content, suggesting that this fungus effectively scavenges essential nutrients and their precursors from nutrient-poor environments.

Transcription patterns of *C. neoformans* isolated directly from the CNS of infected patients were compared with samples incubated *ex vivo* on CSF media (Chen et al. 2014). Carbohydrate importers and the sodium transporter Ena1 are highly induced in both conditions. Interestingly, the inositol transporter gene family is specifically required for *C. neoformans* penetration of the blood brain barrier (Liu et al. 2013). Inositol is present in high concentration in the brain, suggesting an interesting potential targeting mechanisms for this neuropathogen to the CNS (Liu et al. 2013). Additionally, the alkaline-responsive Rim101 transcription factor was also highly induced during CNS infection. Together, these results suggest that *C. neoformans* must actively adapt to host-specific signals while growing in the CNS, including nutrient deprivation and host pH.

Several lines of evidence implicate a role for laccase activity during CNS infections. First, laccase mutants are highly attenuated for virulence in animal models of cryptococcal infection (Noverr et al. 2004). Also, melanised forms of *C. neoformans* can be isolated from CNS tissue during infection (Nosanchuk et al. 2000). The transcript levels of cryptococcal laccases are specifically induced by glucose deprivation, a condition known to be present in the CNS (Williamson 1994). Additionally, the substrates for cryptococcal melanin formation include diphenolic compounds such as epinephrine, DOPA, and norepinephrine (Williamson et al. 1998). The enhanced availability of these diphenolic neurotransmitter molecules in neural tissue has been postulated to be one reason for the neurotropism of this microorganism.

## SUMMARY

*Cryptococcus neoformans* continues to be a significant pathogen among immunocompromised individuals, especially those with advanced HIV infection. As an environmental fungus, this organism has adapted many strategies to survive its trip to disease in the mammalian host. Suggested to have acquired many of its virulence traits from environmental encounters, *C. neoformans* has been referred to as an “accidental pathogen”.

Beginning its journey in the environment, this fungus can interact and infect many soil microbes, and during its interaction with these microbes, *C. neoformans* utilises many of its classical virulence attributes. This fungus is introduced into the mammalian host through the inhalation of spores or desiccated yeast. With advanced methods of spore isolation recently described, the role of spores at this initial step has been able to be elucidated more clearly. However, future work will be required to characterise the innate immune responses to these infectious propagules, and how these responses direct the development of disease.

As *C. neoformans* is inhaled into the mammalian lung, it must adapt to a number of additional stresses including high temperature, increased pH, and changes in essential nutrients and metal concentrations. Ongoing work continues to identify novel upstream and downstream components of the conserved signalling pathways controlling responses to these stresses. This fungus has a dynamic relationship with host phagocytes, in which it actively avoids detection and killing by these cells, but it also requires them for effective CNS dissemination. Inside the host, *C. neoformans* has also developed ways to alter its morphology in order to facilitate survival, including the production of polysaccharide capsule, titan cell formation, and cell wall rearrangement. Continued efforts to understand this delicate host-pathogen interface will be needed to drive the development of novel methods to direct this response in favour of the host.

Finally, in order to effectively finish its journey to the CNS, this fungus has the ability to traverse the BBB through various means. These include direct traversal through endothelial cells, manipulation of the tight junctions of the BBB, and the “Trojan-horse” mechanism. A greater understanding of how *C. neoformans* utilises these different means *in vivo* will provide a path forward for developing new therapeutic targets to control brain invasion.
